# Liver Transplantation Following Immune Checkpoint Inhibitor Therapy: What Do We Need to Know from Clinical and Immunological Perspective?

**DOI:** 10.3390/ijms27062680

**Published:** 2026-03-15

**Authors:** Hee Sun Cho, Soon Kyu Lee

**Affiliations:** 1 Department of Internal Medicine, Division of Hepatology, Seoul St. Mary’s Hospital, College of Medicine, The Catholic University of Korea, Seoul 06591, Republic of Korea; jhs-cho@catholic.ac.kr; 2 The Catholic University Liver Research Center, College of Medicine, The Catholic University of Korea, Seoul 06591, Republic of Korea; 3 Department of Internal Medicine, Incheon St. Mary’s Hospital, College of Medicine, The Catholic University of Korea, Seoul 06591, Republic of Korea

**Keywords:** hepatocellular carcinoma, liver transplantation, immune checkpoint inhibitors, rejection, T cell

## Abstract

Immune checkpoint inhibitors (ICIs) have transformed the therapeutic landscape of advanced hepatocellular carcinoma (HCC), establishing immunotherapy-based combinations as the standard of care. Improved treatment responses have expanded liver transplant eligibility for selected patients with advanced HCC through downstaging or bridging strategies. Such advances have directly influenced transplant candidacy and post-transplant outcomes. However, accumulating evidence indicates that pretransplant exposure to ICIs may disrupt post-transplant immune homeostasis, increasing the risk of acute allograft rejection and graft failure requiring retransplantation. From an immunological perspective, rejection following pretransplant ICI therapy predominantly manifests as T cell-mediated rejection and is characterized by the sustained activation of effector T cells and impairment of regulatory immune pathways. Blockade of immune checkpoint signaling interferes with mechanisms critical for allograft tolerance, including T cell apoptosis and regulatory T cell induction. Recent studies further underscore the importance of the washout period between ICI discontinuation and LT, with longer washout intervals being associated with lower rejection rates. Importantly, timely recognition and appropriate immunosuppressive management can often resolve acute rejection without adversely affecting long-term graft outcomes. This review integrates current immunological insights with emerging clinical evidence to inform optimal transplant timing and management strategies for liver transplant candidates receiving ICIs.

## 1. Introduction

Hepatocellular carcinoma (HCC) is a globally challenging disease, being the fourth-leading cause of cancer-related death. It contributes 80% of all liver cancer cases worldwide. The main etiologies of HCC have been related to viral infection, such as hepatitis B virus and hepatitis C virus. However non-viral causes are expanding, as the prevalence of metabolic dysfunction-associated steatotic liver disease (MASLD) is rapidly increasing [1,2]. The incidence of HCC appears to have plateaued, with steady increases in Western countries offsetting the declines in East and Southeast Asia [3]. As the incidence and mortality attributed to HCC are comparable, this suggests a high case fatality rate and highlights its poor prognosis [1]. Despite advances in surgical and locoregional therapies (LRT) for early HCC, approximately 50–60% of patients are treated with systemic therapies. Tyrosine kinase inhibitors (TKIs), such as sorafenib, lenvatinib, regorafenib, and cabozantinib, have established the backbone of systemic therapy for advanced HCC since 2007 [4]. Recently, the advent of immune checkpoint inhibitors (ICIs) has significantly shifted the therapeutic landscape of advanced HCC toward immunotherapy-based strategies.

The first notable step was the combination of atezolizumab, a programmed death ligand 1 (PD-L1) inhibitor, and bevacizumab, a vascular endothelial growth factor (VEGF) inhibitor, which demonstrated significantly improved overall survival (OS) and progression-free survival (PFS) compared to sorafenib in the IMbrave150 trial, establishing a new first-line standard for unresectable HCC [5]. Another combination of a PD-L1 inhibitor and a cytotoxic T lymphocyte-associated antigen 4 (CTLA-4) inhibitor, durvalumab plus tremelimumab (Durva/Tre), showed statistically improved OS compared to sorafenib [6]. In the updated IMbrave150 trial, atezolizumab plus bevacizumab (Ate/Beva) demonstrated an objective response rate (ORR) of 29.8%, with a complete response (CR) rate of 7.7% and a partial response (PR) rate of 22.1% [7]. Meanwhile, the STRIDE regimen of Durva/Tre achieved an ORR of 20.1%, with a CR rate of 3.1% and a PR rate of 17.0% [6]. Consistent with these findings, the real-world data reported comparable clinical outcomes of Ate/Beva and Durva/Tre regimens [8,9]. Furthermore, in the CheckMate 9DW trial, nivolumab plus ipilimumab (Nivo/Ipi) treatment—a combination of programmed cell death-1 (PD-1) inhibitor and CTLA-4 inhibitor—also demonstrated improved OS compared with lenvatinib or sorafenib in unresectable HCC (hazard ratio, 0.79; 95% confidence interval, 0.65–0.96; *p* = 0.018) [10].

Liver transplantation (LT) is considered a curative therapy for HCC, particularly for early-stage disease within the Milan criteria or other relevant LT criteria [11]. However, most patients exceed these criteria at the time of diagnosis, rendering them ineligible for the surgery. Global efforts have been made to expand the criteria or increase the pool of transplant candidates through effective downstaging strategies [12]. With the emergence of ICIs demonstrating improved treatment response, an increasing number of patients are now meeting eligibility criteria for LT [13]. At the same time, accumulating evidence has raised concerns regarding immune-related adverse events (irAEs) and allograft rejection, complicating post-transplant outcomes [14,15]. In particular, the rate of rejection after LT in patients who received ICI therapy in the pretransplant setting has been reported to be approximately 30%, with a rejection-related mortality rate of 6%. Mechanistically, immune hyperactivation following ICI therapy—characterized by activated recipient T cells—is thought to contribute to the development of rejection after LT [16,17]. Considering this high risk of rejection and rejection-related mortality, careful consideration is warranted when administering ICIs to patients undergoing downstaging or bridging therapies for LT.

In this review, we outline the immunological mechanisms of ICIs within the tumor immune microenvironments (TIME) in advanced HCC, with particular emphasis on currently approved first-line ICI-based therapies and their modulation of T cell–mediated immune responses. We further examine key immunological aspects related to tolerance and the risk of allograft rejection following pretransplant ICI exposure. Finally, we discuss current evidence and clinical strategies for preventing and managing of ICI-associated acute rejection after liver transplantation to improve clinical outcomes in the evolving immunotherapy era. A literature search was conducted in PubMed/MEDLINE, Google Scholar, and Web of Science to identify studies published within the past 10 years on immune checkpoint inhibitors for HCC and liver transplantation, with a focus on cellular and molecular immunological mechanisms, rejection pathways, and related prevention and management strategies.

## 2. Immune Landscape of HCC in the Context of Immunotherapy

### 2.1. Immunology in Hepatocarcinogenesis

HCC mainly develops from chronic liver inflammation, which eventually progresses to liver cirrhosis [18]. The liver maintains its unique tolerogenic immune microenvironment, composed of diverse immune cell populations, in order to endure gut-derived microbial signals. Immune effector cells that contribute to antitumor response include CD8^+^ T cells, CD4^+^ T cells, natural killer (NK) cells, dendritic cells (DCs), and B cells [19]. The infiltration of CD8^+^ T cells in HCC primarily induces higher immunogenicity by presenting tumor-specific antigens [20]. In contrast, immune suppressor cells—such as regulatory T cells (Tregs), myeloid-derived suppressor cells (MDSCs), and tumor-associated macrophages (TAMs)—inhibit the function and proliferation of effector cells, thereby promoting immune tolerance and facilitating tumor immune escape [19]. An imbalance between immune effector and suppressor cells, driven by the chronic inflammation, is considered as a major mechanism underlying hepatocarcinogenesis [18].

One of the key strategies by which immune suppressor cells operate is the reprogramming of immune effector cells into immune-tolerant phenotypes, such as exhausted CD8^+^ T or NK cells, regulatory DCs (DCregs), and regulatory B cells (Bregs), thereby dampening antitumor immunity [19]. CD8^+^ T cell activation is suppressed by PD-L1 expressed on tumor cells, DCregs, and MDSCs. Tregs and TAMs secrete immunosuppressive cytokines such as interleukin-10 (IL-10) and transforming growth factor-β (TGF-β), which inhibit the activation and proliferation of CD8^+^ T cells. Functionally impaired, exhausted CD8^+^ T cells overexpress inhibitory receptors including PD-1, CTLA-4, T cell immunoglobulin and mucin-domain containing protein-3 (TIM-3), lymphocyte activation gene-3 (LAG-3) and T cell immunoreceptor with Ig and ITIM domains (TIGIT), further deepening immune escape [8,21,22]. NK cells also undergo functional exhaustion upon overstimulation by tumor cells or damaged self-cells [23]. These exhausted NK cells express PD-L1 and contribute to immune response suppression [19]. Tregs also inhibit DC maturation by secreting cytokines including IL-10, TGF-β, and VEGF, and they induce PD-L1 expression on DCs, thereby promoting conversion into DCregs. DCregs, in turn, secrete high levels of IL-10 and contribute to the establishment of tolerogenic immune environment [24]. B cells can also differentiate into Bregs in response to various Toll-like receptor (TLR) ligands and cytokines. Notably, the binding of CD40L expressed by T cells to CD40 on B cells serves as a pivotal signal for Breg induction [25]. Overall, a shift in the immune landscape from effector to suppressor cells increases the proportion of suppressor cells and diminishes the overall antitumor activity of immune cells. In parallel, the expression of cytokines and growth factors that promote tumor cell proliferation or inhibit apoptosis is increased, ultimately facilitating hepatocarcinogenesis [18]. Targeting these immune checkpoints expressed on immune cells is a main strategy in immunotherapy.

### 2.2. Synergistic Mechanism of Anti-PD-L1 and Anti-VEGF

Several studies have demonstrated the synergistic antitumor efficacy of combining ICIs with anti-VEGF receptor agents [26,27,28]. In the Barcelona Clinic Liver Cancer (BCLC) guideline updated in 2025, a major change was made in the systemic treatment recommendations for advanced HCC (BCLC stage C), with Ate/Beva, Durva/Tre, and Nivo/Ipi becoming the first-line therapies [29].

Atezolizumab and durvalumab are monoclonal antibodies against PD-L1, while nivolumab is a monoclonal antibody against PD-1 [30]. PD-L1 interacts with PD-1 receptor on effector CD8^+^ T cells. This interaction recruits the phosphatase SHP2, leading to dephosphorylation of proximal T cell receptor (TCR) signaling molecules and blocking the TCR-induced stop signal. Disruption of TCR-induced stop signals leads to a lack of interaction time with T cells and antigen-presenting cells (APCs), thus resulting in unstable immunological synapse formation between them. Ultimately, insufficient T cell activation results in immune-exhausted status [31,32,33,34]. PD-1-expressing DCs also disrupt CD8^+^ T cell function [35]. In a chronic environment with sustained antigen exposure such as infection or tumor conditions, persistent PD-1 stimulation leads to a state of exhaustion or anergy in CD8^+^ T cells. Exhausted T cells exhibit impaired effector function and reduced proliferative capacity [36]. In B cells and NK cells, PD-l signaling acts as an inhibitory regulator of their activity [37,38]. Moreover, the PD-1/PD-L1 pathway plays a pivotal role in converting naïve CD4^+^ T cells into induced Tregs and maintaining their suppressive functions to keep the peripheral immune tolerance [39]. Taken together, blockade of PD-1 signaling restores the immunologic function of effector cells and enhances antitumor responses.

VEGF is an endothelial cell mitogen affecting angiogenesis and abnormal vascular permeability, which in turn limits the infiltration of effector immune cells. Angiogenesis-mediated down-regulation of endothelial adhesion molecules induces endothelial cell anergy, characterized by unresponsiveness to inflammatory cytokines, thereby impairing the expression of adhesion molecules and the infiltration of cytotoxic immune cells into tumors [40,41,42]. Furthermore, VEGF-induced immature DCs exhibit incomplete antigen-presenting function, thereby hindering T cell activation [43]. In parallel, VEGF promotes the migration of immunosuppressive cells (MDSCs, TAMs and Tregs) into the TIME and enhances their infiltration, leading to the secretion of inhibitory cytokines and suppression of antitumor immunity [44,45]. Accordingly, monoclonal antibodies targeting VEGF inhibit tumor angiogenesis, promote vascular normalization, reverse endothelial anergy, and restore the pro-immune environment. Overall, the blockade of VEGF pathway results in the enhanced delivery of therapeutic agents and cytotoxic immune cells into the tumor [41,42]. Collectively, the combination of anti-PD-L1 antibodies and VEGF inhibitors, as represented by Ate/Beva, enhances antitumor immunity by reshaping the TIME, resulting in increased effector cell infiltration and reduced immune suppression in HCC [46].

### 2.3. Synergistic Mechanism of Anti-PD-L1 and Anti-CTLA-4

The other first-line systemic therapies for advanced-stage HCC are Durva/Tre and Nivo/Ipi. While durvalumab and nivolumab target the PD-(L)1 pathway, tremelimumab and ipilimumab are monoclonal antibodies targeting CTLA-4, a key inhibitory receptor involved in early T cell activation. At the early stage of T cell activation, CD28 expressed on naive or resting T cells binds to CD80 (B7-1) or CD86 (B7-2) ligands on APCs, delivering co-stimulatory signals essential for T cell activation in lymphoid organs. In contrast, CTLA-4 is expressed only on activated T cells and also binds to CD80 or CD86, but with significantly higher affinity than CD28. By outcompeting CD28, CTLA-4 dampens T cell activation. The balance between CD28 and CTLA-4 signaling determines overall T cell activation during the priming phase [47,48,49]. CTLA-4 also inhibits T cell activation in a signaling-independent manner by sequestrating CD80 and CD86, thereby preventing their interaction with CD28, a process known as trans-endocytosis [50]. Consequently, CTLA-4 suppresses CD8^+^ T cell cytotoxicity and impairs CD4^+^ T cell-driven activation of cytotoxic T lymphocytes, B cells, and NK cells [51].

While CTLA-4 modulates the early priming stages of naive T cell activation in lymphoid organs, PD-(L)1 blockade primarily acts at later effector phases to retain activated T cells within peripheral tissues and TIME. CTLA-4 functions exclusively on T cells, whereas the PD-(L)1 pathway exerts regulatory effects on a broader spectrum of immune cells, including B cells, NK cells, DCs and monocytes [49,52]. Effective immunotherapy requires strategies that can broadly interfere with the immune evasion mechanisms and target multiple stages of cancer immune escape. To maximize the efficacy, it is essential to optimize the timing and sequencing of specific combinations of targeted agents and immunotherapies [53]. Consistent with this, combinations of Durva/Tre or Nivo/Ipi have shown improved OS and comparable safety profile compared with monotherapies [6,10,54].

## 3. Immunotherapy Before LT

Surgical resection and liver transplantation are considered as curative options; however, only 30–40% of patients are diagnosed within the Milan criteria, leaving the majority ineligible for surgery [55]. Even among patients within the Milan criteria, LRT or systemic bridging therapies are often required while awaiting an adequate donor. For those who fall outside the criteria, more aggressive downstaging strategies are necessary, and efforts have been made to expand liver transplant eligibility. Commonly used LRTs include transcatheter arterial chemoembolization (TACE), transarterial radioembolization (TARE), ablation, and stereotactic body radiation therapy (SBRT); no single modality has demonstrated clear superiority over others [56]. A study reported effective downstaging in 17 patients initially outside the Milan criteria treated with Ate/Beva combined with LRT—including Y-90 radioembolization (n = 6), TACE (n = 4), ablation (n = 2), and SBRT (n = 1)—resulting in a 97% objective response rate and successful downstaging to within the Milan criteria in 13 patients [57]. In a multiregional US study of 117 HCC patients, 94% of whom received concurrent ICIs and LRTs including TARE, TACE, and ablation, 75.6% were successfully downstaged within a median of 5.6 months, with a 3-year post-LT survival of 85% [58]. Taken together, these studies suggest an optimistic future where the combination of ICIs and multimodal LRT may facilitate effective downstaging through synergistic cytoreductive and immunomodulatory effects, potentially increasing transplant eligibility. Extended LT criteria—such as the Up-to-Seven, UCSF, MetroTicket 2.0, MORAL, and NYCA criteria—have been proposed, and several studies reported outcomes comparable to those of the Milan criteria in selected populations. The Up-to-Seven criteria allow transplantation when the sum of the size of the largest tumor and the number of tumors does not exceed seven, while the UCSF criteria permit a single tumor ≤6.5 cm or up to three tumors each ≤4.5 cm with a total diameter ≤8 cm. The Metroticket 2.0 incorporates tumor number, size, and alpha-fetoprotein (AFP) levels [59]. The NYCA criteria integrate tumor size and number together with AFP dynamics, and response to LRT to refine candidate selection [60]. Beyond conventional criteria, emerging biomarkers such as AFP-L3, des-gamma-carboxyprothrombin (DCP), and circulating tumor DNA (ctDNA) have been introduced to predict post-transplant HCC recurrence. Composite scoring models such as the GALAD score—integrating sex, age, AFP, AFP-L3, and DCP—and the MoRAL score—combining pretransplant AFP and PIVKA-II levels—have been further proposed to enhance recurrence prediction and candidate stratification [59]. However, no validated biomarker currently exists to guide pretransplant ICI use or to stratify rejection risk in this specific context. Notably, T cell activation markers—including CD4^+^ and CD8^+^ T cell counts, which are established indicators of acute allograft rejection—have been reported at low levels even in patients who developed acute rejection following pretransplant ICI therapy, suggesting that these markers may similarly serve as potential biomarkers for rejection surveillance in the context of prior ICI exposure [61]. With the advent of artificial intelligence, radiomics-based imaging biomarkers are also being actively investigated [62]. The emergence of ICI combination therapy, which modulates key steps of the immune mechanisms, has led to improved treatment responses in inoperable advance-stage HCC compared with previous TKI regimens [5,6]. In this context, growing evidence supports the use of ICI-based therapies, with or without LRT, as a bridging or downstaging strategies in pretransplant patients.

Unlike other solid organs, liver has a unique immune-tolerant background involving various immune cells, resulting in T cell tolerance, exhaustion, or apoptosis. This feature contributes to lower rates of graft rejection and improved long-term graft survival compared with the transplants of other solid organs. In terms of immunosuppressant treatment, the required maintenance dose is generally lower and complete treatment withdrawal may be possible in selected long-term recipients [63,64]. Nevertheless, acute allograft rejection occurs in 20–40% of recipients, and its occurrence is associated with increased overall mortality and, in some cases, may lead to graft failure requiring retransplantation [65,66]. Given that ICI-based therapies activate immune responses, pretransplant ICI use may exert conflicting effects on the immune system, thereby increasing the risk of allograft rejection and graft failure.

### 3.1. Mechanisms of Liver Tolerance and Rejection Following Pretransplant Immune Checkpoint Inhibitor Therapy

In the context of LT, several mechanisms have been proposed to explain its tolerogenicity, including the release of soluble donor major histocompatibility complex (MHC) class I molecules, the high-load antigen effect, and the presence of donor-derived passenger leukocytes (PLs) [67,68]. Donor liver grafts release large amounts of soluble MHC class I molecules without co-stimulatory signals, leading to apoptosis of effector T cells and thereby promoting graft survival with reduced rejection [69]. Additionally, the high load of these MHC molecules and cytokines, derived from the graft’s substantial volume, can dilute and deplete the recipient’s T cells through apoptosis [70]. Donor-derived PLs circulate in the recipient’s lymphoid tissues, and their sustained presence contributes to immune tolerance by modulating the host immune response [68].

In addition, several immune cells—Tregs, DCs, MDSCs, Kupffer cells (KCs), liver sinusoidal endothelial cells (LESCs) and hepatic stellate cell (HSCs)—contribute to the establishment of tolerance. Specifically, Tregs play pivotal roles in achieving tolerance through direct secretion of IL-10, TGF-β, granzyme B, and perforin (direct mechanisms). Moreover, the expression of CD39/CD73, resulting in ATP depletion, and CD25-dependent apoptosis are also well-known mechanisms of Treg-mediated immune suppression (indirect mechanisms) [71,72]. DCs exhibit upregulation of PD-L1 and IL-10 secretion, thereby promoting the differentiation of Foxp3^+^ Tregs [73,74]. KCs also secrete anti-inflammatory cytokines such as IL-10, TGF-β, and nitric oxide, ultimately inducing immune tolerance by inhibiting T cell proliferation [68,75]. LESCs and MDSCs suppress Th1 and Th17 responses through IL-10 and PD-1 signaling and facilitate the differentiation of naive T cells into Tregs, leading to T cell tolerance [68,76]. With growing interest in the gut–liver axis, recent studies have also demonstrated that gut microbiota-derived metabolites affect LT rejection, mainly by modulating immune tolerance through regulation of Treg and Th17 cell populations [71,77,78,79].

In the transplantation setting, the induction of immune tolerance is closely linked to early inflammatory responses [67]. Notably, interferon-γ (IFN-γ), which is transiently elevated during ischemic-reperfusion injury, has been shown to upregulate PD-L1 expression on LSECs and MDSCs uniquely in post-LT environment, thereby contributing to the establishment of allograft tolerance [80]. In the early post-LT state, Foxp3^+^CD25^+^CD4^+^ Tregs are markedly increased in the liver graft and recipient spleen, and they express high levels of CTLA-4, which plays a critical role in inducing and maintaining LT tolerance [81].

**Figure 1:**
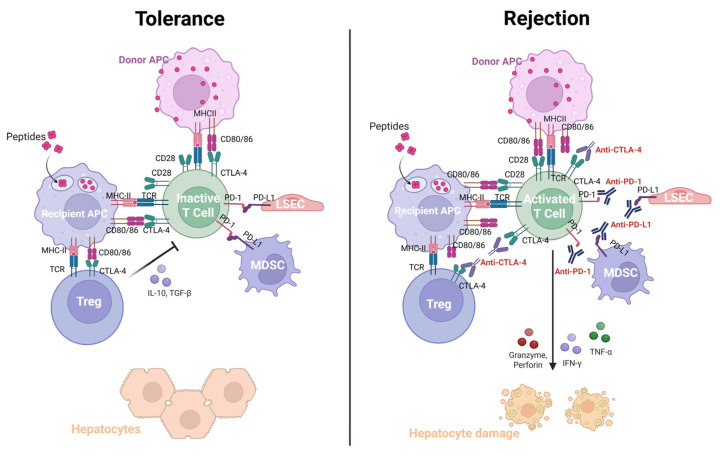
Immunological mechanism of tolerance and rejection after immune checkpoint inhibitors in liver transplantation. Schematic illustration of immune tolerance and rejection in liver transplantation after immune checkpoint inhibitor therapy, highlighting checkpoint signaling, effector and regulatory T cell responses, and cytokine production within the liver graft. APC, antigen presenting cells; IFN, interferon; LSEC, liver sinusoidal endothelial cells; MDSC, myeloid-derived suppressor cells; TNF-α, tumor necrosis factor-α; Treg, regulatory T cells.

In the context of rejection, recognition of donor alloantigens expressed on donor or recipient APCs by recipient T cells initiates of T cell-mediated rejection (TCMR) in LT [82]. In contrast to the tolerogenic state of LT, blockade of PD-L1 and CTLA-4 signaling following ICI therapy leads to reduced T cell apoptosis and impaired induction of Tregs, resulting in increased infiltration of effector T cells within the graft liver [16,71]. This shift toward enhanced systemic and intrahepatic T cell proliferation, accompanied by increased pro-inflammatory cytokine production, ultimately triggers immune responses against the donor liver. Consistently, several studies have shown that CTLA-4 blockade in the post-transplant setting is associated with increased T cell proliferation through inhibition of T cell apoptosis, thereby inducing acute rejection. In accordance with these observations, compared with immunotolerant recipients, patients with acute rejection had reduced expression of PD-1 and CTLA-4 on CD8^+^ and CD4^+^ T cells, suggesting increased inflammatory responses within the graft liver [83,84]. Indeed, activated CD8^+^ T cells secreting tumor necrosis factor-α (TNF-α), IFN-γ, granzyme, and perforin, as well as CD4^+^ Th1 and Th17 cells promote TCMR in LT patients [85,86]. The immunological mechanisms underlying liver allograft tolerance and rejection following immune checkpoint inhibitor therapy are summarized in Figure 1.

Based on these immunological mechanisms, the rejection rate among patients receiving pretransplant ICI therapy has been reported to be approximately 30%. Moreover, the rejection-related mortality rate is about 6%, which is substantial in the LT setting [16]. Notably, when ICIs are used in the post-LT setting, the risk of rejection is markedly higher, with graft loss reported in up to 50% of cases despite appropriate rejection treatment including high-dose steroid therapy [87]. Given these high rejection rates, the use of ICIs for recurrent HCC after LT is generally not recommended [88]. Meanwhile, in patients receiving ICI therapy before transplantation, adequate prevention and management of graft rejection are crucial for improving clinical outcomes.

### 3.2. Management and Prevention of Graft Rejection Following Pretransplant Immune Checkpoint Inhibitor Therapy

One of the primary reasons why ICIs influence post-transplant immune tolerance is their prolonged persistence in the recipient. Hence, an adequate washout period between the last ICI administration and LT is a critical factor, given that the serum half-life of ICIs ranges up to 30 days: about 27 days for atezolizumab, 20 days for tremelimumab, and approximately 25 and 15 days for nivolumab and ipilimumab, respectively [89]. However, from a pharmacodynamic perspective, occupancy of ICI pharmacological targets may persist significantly longer. The median receptor occupancy of anti-PD-L1 or anti-PD-1 antibodies on peripheral T cells has been reported to be 60–70% and to persist at that level for more than 2 months despite a single dose, irrespective of dose level [90,91,92]. With multiple infusions, some patients showed prolonged PD-1 occupancy exceeding 50% from the last administration, persisting for over 200 days [90]. In addition, Robert et al. demonstrated that CTLA-4 blockade with tremelimumab induces sustained diversification of the peripheral T cell receptor repertoire that remains evident 30–60 days after treatment, indicating prolonged immunomodulatory effects independent of circulating drug levels [93]. Overall, prolonged pharmacodynamic and immunomodulatory effects of ICIs complicate the determination of an appropriate washout period, which remains debated (Table 1).

In the first reported case of pretransplant ICI use, a patient received nivolumab for nearly 2 years, with the last dose administered 8 days before transplantation. The patient subsequently had fallen into acute graft rejection with hepatic necrosis and died. In this case, nivolumab was administered shortly before transplantation due to repeatedly recurrent HCC [94]. As such, consideration should not be limited to the washout period of monoclonal antibodies alone; the timing should be optimized to ensure sustained tumor control while awaiting LT. Schwacha-Eipper et al. discontinued nivolumab 6 weeks prior to activation on the LT waiting list, and the patient underwent transplantation 15 weeks after discontinuation, with favorable outcomes and without tumor recurrence or graft rejection [95]. In another case, durvalumab was discontinued for 90 days based on the evidence that PD-L1 receptor occupancy was observed for up to 85 days, and no adverse outcome was observed [90,96]. With the combination of nivolumab and ipilimumab, Lizaola-Mayo et al. presented successful LT with 9 weeks of washout period [97]. Immunosuppression intensity and substantial transfusion may affect ICI clearance and immune modulation, as Tabrizian et al. reported that eight patients receiving their last nivolumab dose within 4 weeks experienced no severe rejection or death [98]. Recently, a retrospective multicenter cohort study from China reported that a washout interval of 30 days was independently associated with a reduced risk of graft rejection, with rejection rates of 71.4% in patients with a washout period of less than 30 days versus 12.9% in those with a washout period of 30 days or longer [99]. Similarly, a larger cohort study including 209 patients demonstrated a significantly higher rejection rate when the washout interval was shorter than 30 days [100]. As the first systematic meta-analysis on this topic, Rezaee-Zavareh et al. highlighted that reduced allograft rejection is associated with increased age and longer ICI washout period, with a median washout duration of 94 days. Notably, over 80% of allograft rejection episodes were resolved with medical therapy, with no significant impact on OS [14]. Overall, the literature suggests that a washout interval of 4 weeks to 3 months between the last dose of ICI therapy and LT may be appropriate, although further studies are required to define the optimal timing.

As the PD-1/PD-L1 pathway plays a critical role in downregulating the immune system, studies in post-LT patients treated with ICI have suggested a correlation between allograft PD-L1 expression and the risk of graft rejection. In reported cohorts, all recipients with positive PD-L1 expression in allograft liver showed acute graft rejection, whereas no rejection was observed among those with negative PD-L1 expression. These findings suggest that PD-L1 expression in the graft liver could serve as a potential marker in the context of pretransplant anti PD-L1 therapy [101,102,103]. In some cases, PD-L1 expression in the graft liver was negative before transplantation but became positive after transplantation [94,104]. Accordingly, the assessment of PD-L1 expression via liver biopsy performed before and after transplantation may help predict the risk of acute graft rejection. Further studies are warranted to clarify whether changes in allograft PD-L1 expression represent an adaptive response to perioperative immunosuppression and whether such changes are associated with acute graft rejection.

Timely and optimal medical treatment following a precautionary strategy is essential for favorable outcomes. Management of pretransplant ICI-related allograft rejection varies among centers, but is generally guided by established allograft rejection treatment protocols (Table 2) [105,106]. As the underlying mechanism predominantly involves TCMR, histological grading of liver allograft rejection based on the Banff schema is required to exclude other alternative diagnoses and to grade the severity of rejection. The Banff grading system evaluates three key histopathologic features—portal inflammation, bile duct inflammation and/or damage, and venous endothelial inflammation—to calculate the Rejection Activity Index (RAI), which classifies the severity as mild (RAI ≤ 4) and moderate or severe (RAI ≥ 5) [107]. Both early and late TCMR are managed according to the same severity-based treatment algorithm. Mild TCMR is treated with escalation of calcineurin inhibitor (CNI) dosing, with or without addition of other agents such as low-dose oral prednisone, mycophenolate mofetil (MMF), or mammalian target of rapamycin inhibitors (mTORi); pulse steroid therapy is generally unnecessary in this setting. Moderate to severe TCMR should be managed with pulse steroid therapy, typically given 500–1000 mg of intravenous methylprednisolone daily or every other day for three doses, along with increased maintenance CNI dosing and/or other agents if appropriate. In patients with severe or steroid-resistant TCMR accompanied by significant cholestasis, lymphodepleting antibody therapy may be considered. Several successful cases of antithymocyte globulin (ATG) use have been reported, whereas evidence on the use of alemtuzumab is limited. Steroid-resistant or repeated TCMR may progress to chronic TCMR with irreversible bile duct and/or vascular injury. For chronic rejection, cyclosporine-based regimen should be switched to tacrolimus-based regimen. Although antibody-mediated rejection (AMR) is uncommon, it may coexist with TCMR and should be considered in patients with TCMR not responding to the standard therapy. In such cases, conversion to tacrolimus along with second-line therapies—such as plasmapheresis, intravenous immunoglobulin (IVGV), and B cell or plasma cell depleting agents (e.g., rituximab, bortezomib)—may be required. If graft failure progresses despite medical treatment, retransplantation should be considered [105,106,108,109]. In patients with rapidly progressive HCC or prior irAEs, we suggest securing at least a minimal washout period before transplantation, with approximately 4 weeks considered a realistic threshold based on the half-life of immune checkpoint inhibitors and currently available clinical evidence. In such high-risk patients, a more intensive post-transplant immunosuppressive strategy may also be considered, including higher maintenance doses of CNIs together with additional immunosuppressive agents. However, if clinical circumstances allow, extending the washout period up to 3 months may be considered based on the existing literature.

With regard to allograft PD-L1 expression, current evidence remains insufficient to determine whether it serves as a direct biomarker of acute rejection or instead reflects an adaptive response to perioperative immunological changes. Therefore, at present, PD-L1 expression should be interpreted cautiously and considered only as a supplementary reference marker.

## 4. Conclusions

ICI therapies have emerged as a new standard of care for advanced hepatocellular carcinoma and have expanded the possibility of downstaging, thereby increasing eligibility for liver transplantation. However, pretransplant exposure to ICIs may lead to persistent immune activation after transplantation, resulting in an increased risk of allograft rejection and necessitating careful clinical vigilance. Accumulating evidence suggests that the interval between ICI discontinuation and transplantation, together with appropriate post-transplant medical management, plays a critical role in mitigating the risk of allograft rejection.

However, recent retrospective studies and systematic meta-analyses have included heterogeneous ICI regimens, resulting in the absence of clearly defined optimal washout intervals for specific ICI regimens. Prospective studies evaluating the use of ICIs in the liver transplantation setting are therefore needed. Additional research is also required to clarify the relationship between irAEs and acute rejection, as the presence and severity of irAEs may be associated with post-transplant outcomes. Moreover, the development of an appropriate rejection management algorithm will be important for improving graft outcomes. Addressing these gaps will require further studies to better understand the immunological pathways linking ICI exposure to graft rejection, as well as research aimed at identifying reliable biomarkers. Overall, prospective multicenter studies will be essential to establish evidence-based guidelines that balance oncologic benefit with transplant safety in patients undergoing liver transplantation after ICI therapy.

## Figures and Tables

**Table 1:** Studies reporting washout periods before liver transplantation after immune checkpoint inhibitor treatment.

Authors	Year	Sample Size	Study Design	ICIs	Key Results	Washout Period	Management of Rejection
Nordness et al. [94]	2020	1	Case report	Nivolumab	Acute graft rejection with hepatic necrosis, resulting in death	8 days	High-dose steroids Increased IS dose ATG
Schwacha-Eipper et al. [95]	2020	1	Case report	Nivolumab	No graft rejection or tumor recurrence	6 weeks (actual: 15 weeks)	Not reported
Sogbe et al. [96]	2021	1	Letter to editor	Durvalumab	No graft rejection or tumor recurrence in 2-year follow-up	90 days (actual: 4 months)	Not reported
Lizaola-Mayo et al. [97]	2021	1	Correspondence	Nivolumab Ipilimumab	No graft rejection or tumor recurrence within 6 months	8 weeks (actual: 9 weeks)	Increased IS dose ATG
Tabrizian et al. [98]	2021	9	Letter to editor	Nivolumab	Eight patients without rejection. One patients with low tacrolimus level developed acute rejection	≤30 days (8 patients) 253 days (1 patient)	Increased IS dose
Guo et al. [99]	2024	83	Retrospective multicenter	Pembrolizumab Atezolizumab Camrelizumab Sintilimab Tislelizumab Nivolumab	Acute rejection in 23 patients (3 deaths)	≥30 days	High-dose steroid IVGV or ATG Increased IS dose Plasma exchange
Fang et al. [100]	2025	209	Retrospective multicenter	Pembrolizumab Atezolizumab Camrelizumab Sintilimab Tislelizumab	Acute rejection in 36 patients; risk increased with washout < 30 days and presence of irAEs	≥30 days.	Not reported
Rezaee-Zavareh et al. [14]	2025	91	Systemic meta-analysis	Pembrolizumab Atezolizumab Camrelizumab Sintilimab Nivolumab Durvalumab	Graft rejection in 24 patients; risk decreased with longer washout period and older age	≥94 days	Not reported

ICIs, immune checkpoint inhibitors; IS, immunosuppressive agent; ATG, antithymocyte globulin; IVGV, intravenous immunoglobulin; irAEs, immune-related adverse events.

**Table 2:** Management of allograft rejection following pretransplant immune checkpoint inhibitor exposure.

Type of Rejection	Recommended Treatment	Specific Regimen
Mild rejection	Increase maintenance CNI dose	
	Consider other immunosuppressive agent	Low-dose oral prednisone, MMF, mTORi
Moderate to severe rejection	High-dose pulse steroid therapy	IV MPD 500–1000 mg daily or every other day × 3 doses
	Increase maintenance CNI dose	
	Consider other immunosuppressive agent	Low-dose oral prednisone, MMF, mTORi
Severe rejection with cholestasis	Consider lymphodepleting antibody therapy	ATG, alemtuzumab
Steroid-resistant rejection	Consider lymphodepleting antibody therapy	ATG, alemtuzumab
	Consider antibody depleting therapy	Plasmapheresis, IVIG, rituximab, bortezomib
Chronic rejection	Convert cyclosporine to tacrolimus	

CNI, calcineurin inhibitors; MMF, mycophenolate mofetil; mTORi, mTOR inhibitor; MPD, methylprednisolone; ATG, antithymocyte globulin; IVIG, intravenous immunoglobulin.

## Data Availability

No new data were created or analyzed in this study. Data sharing is not applicable to this article.

## Abbreviations

The following abbreviations are used in this manuscript:

AFP	Alpha-fetoprotein
AMR	Antibody-mediated rejection
APC	Antigen-presenting cell
ATG	antithymocyte globulin
Ate/Beva	Atezolizumab plus bevacizumab
BCLC	Barcelona Clinic Liver Cancer
Breg	Regulatory B cell
CNI	Calcineurin inhibitor
CR	Complete response
CTLA-4	Cytotoxic T lymphocyte–associated antigen 4
DC	Dendritic cell
DCreg	Regulatory dendritic cell
Durva/Tre	Durvalumab plus tremelimumab
HCC	Hepatocellular carcinoma
HBV	Hepatitis B virus
ICI	Immune checkpoint inhibitor
IFN-γ	Interferon gamma
IL	Interleukin
irAE	Immune-related adverse event
KC	Kupffer cell
LAG-3	Lymphocyte activation gene 3
LESC	Liver sinusoidal endothelial cell
LRT	Locoregional therapies
LT	Liver transplantation
MASLD	Metabolic dysfunction–associated steatotic liver disease
MDSC	Myeloid-derived suppressor cell
MHC	Major histocompatibility complex
MMF	Mycophenolate mofetil
mTORi	Mammalian target of rapamycin inhibitor
NK cell	Natural killer cell
Nivo/Ipi	Nivolumab plus ipilimumab
ORR	Objective response rate
OS	Overall survival
PD-1	Programmed cell death 1
PD-L1	Programmed death ligand 1
PFS	Progression-free survival
PL	Passenger leukocyte
PR	Partial response
RAI	Rejection activity index
SBRT	Stereotactic body radiation therapy
TACE	Transcatheter arterial chemoembolization
TAM	Tumor-associated macrophages
TARE	Transarterial radioembolization
TCMR	T cell-mediated rejection
TCR	T cell receptor
TGF-β	Transforming growth factor beta
TIGIT	T cell immunoreceptor with Ig and ITIM domains
TIM-3	T cell immunoglobulin and mucin-domain containing protein-3
TIME	Tumor immune microenvironment
TKI	Tyrosine kinase inhibitor
TLR	Toll-like receptor
TNF-α	Tumor necrosis factor alpha
Treg	Regulatory T cell
VEGF	Vascular endothelial growth factor
